# Response to Kunos et al. and Lotersztajn and Mallat

**DOI:** 10.1172/JCI156247

**Published:** 2022-01-04

**Authors:** Simeng Wang, Qingzhang Zhu, Guosheng Liang, Tania Franks, Magalie Boucher, Kendra K. Bence, Mingjian Lu, Carlos M. Castorena, Shangang Zhao, Joel K. Elmquist, Philipp E. Scherer, Jay D. Horton

**Affiliations:** 1 Department of Internal Medicine and; 2 Department of Molecular Genetics, University of Texas Southwestern Medical Center, Dallas, Texas, USA.; 3 Drug Safety Research and Development, Pfizer Inc., Groton, Connecticut, and Cambridge, Massachusetts, USA.; 4 Internal Medicine Research Unit, Pfizer Worldwide Research, Development, and Medical, Cambridge, Massachusetts, USA.

**Keywords:** Metabolism, Fibrosis, Insulin signaling, Obesity

## The authors reply:

Kunos et al. (1) raise several issues that we can further clarify. We acknowledge that we could not use the TD97070 diet, which contains Primex hydrogenated vegetable shortening, a *trans* fat–rich mix that was discontinued in 2018. Therefore, we used the D12492 diet. The fat content in TD97070 and D12492 is 33.5% and 34.9% of calories, with a saturated fat content of 45% and 32%, respectively. A possible more important difference is that TD97070 contains 24% *trans* fats. Nevertheless, the cited mechanism by which the high-fat diet (HFD) increases anandamide (AEA) is that monounsaturated fatty acids (MUFAs) generated via SCD1 activity (but not diet-derived MUFAs) function as endogenous fatty acid amide hydrolase inhibitors mediating HFD-induced increases in hepatic AEA, which then activates hepatic cannabinoid receptor 1 (CB-1) to induce insulin resistance (2). This is precisely why we also carried out studies using a high-sucrose diet to maximally induce SREBP-1c, SCD1, and MUFA synthesis (3, 4). Despite induction of SCD1, we found no differences between *Cnr1^fl/fl^* and *Hep-Cnr1^–/–^* mice (Figure 2 in ref. 5). This mechanism is also operational in insulin-resistant states, which leads to activation of SCD1 and MUFA synthesis (6), yet, in our study, did not induce CB-1.

Regarding the genetic background, our *Cnr1^fl/fl^* mice were generated on a C57BL/6N background and backcrossed with C57BL/6J mice for six generations. Given the potentially different mixtures of 6N and 6J strains used by the various research groups, it is impossible to exclude subtle genetic differences that might contribute to phenotypic differences.

We agree that a rescue model of CB-1 reexpressed in hepatocytes of *Cnr1^–/–^* mice would be ideal. However, the available liver-specific promoters would massively overexpress CB-1 compared with the extremely low physiological levels of CB-1 expression in hepatocytes. The overexpression would preclude reaching any firm conclusions.

Finally, it was stated that we did not cite any human or animal studies consistent with the role of CB-1 in insulin resistance. Relevant references were included in which there were no changes in body weights (refs. 25–30 in our study).

In response to Lotersztajn and Mallat (7), it is true that we relied on CB-1 mRNA and not protein measurements. We attempted to measure CB-1 protein in liver membranes using three different commercially available antibodies but were unsuccessful, despite detecting CB-1 in extracts from hypothalami. An additional issue raised was that the single-cell RNA-Seq (scRNA-Seq) was performed in livers of HFD-fed mice, a model, they state, in which hepatic stellate cells (HSCs) “are hardly activated.” The purpose of the scRNA-Seq study was to ensure that we were not overlooking a cell type in which CB-1 became highly expressed in response to the diets. The CCl_4_ studies were used to maximally increase stellate cell activation and induce fibrosis, which occurred to an identical extent in WT and *Hsc-Cnr1^–/–^* mice. Since the primary purpose was to determine whether deleting CB-1 in HSCs alters the development of fibrosis, we did not further investigate CB-1 signaling. We agree that fibrosis is a complex process involving multiple cell types in liver. Our data only show that there was no clear contribution of CB-1 in hepatocytes and HSCs to the development of fibrosis or insulin resistance.

## Version 1 01/04/2022

Electronic publication
